# Structures of the reaction products of lithium metal with *N*,*N*-di­methyl­acetamide and tetra­methyl­urea

**DOI:** 10.1107/S2056989026009989

**Published:** 2026-10-30

**Authors:** Andrew Purdy, Raymond J. Butcher

**Affiliations:** a Naval Research Laboratory, Chemistry Division, Code 6100, 4555 Overlook Av, SW, Washington DC 20375, USA; b Department of Chemistry Howard University, 525 College Street NW Washington DC 20059 USA; University of Buenos Aires, Argentina

**Keywords:** crystal structure, β-diketonate, Li reactivity, alkoxide

## Abstract

The reaction of lithium metal with dry *N*,*N*-di­methyl­acetamide produced crystals of *catena*-poly[(μ_3_-*N*,*N*-di­methyl­acetoacetamidato)lithium(I)], [Li(C_6_H_10_NO_2_)]_*n*_, (**I**), which crystallizes as a coordination polymer with a chain of fused Li_2_O_2_ rings. A similar reaction of lithium metal with *N*,*N*-tetra­methyl­urea produces crystals of hexa­kis­[μ_3_-1,2-bis­(di­methyl­amino)-2-oxo­ethan-1-olato]hexa­lithium(I), [Li_6_(C_6_H_13_N_2_O_2_)_6_], (**II**), which crystallizes as a hexa­mer, with the alkoxide O atoms in a Li_6_O_6_ barrel-shaped cluster with threefold symmetry, and the keto O atoms bonded to the lithium atoms outside the cage.

## Abstract 2

The reaction of lithium metal with dry *N*,*N*-di­methyl­acetamide and *N*,*N*-tetra­methyl­urea produced crystals of the lithium *N*,*N*-di­methyl­acetoacetamidate and *N*,*N*-tetra­methyl­urea produces crystals of the lithium bis-1,2-(*N*,*N*-di­methyl­amino)­ethan-1-one-2-olate.

## 1. Chemical context

Even though highly polar solvents containing carbonyl groups like propyl­ene carbonate are generally believed to be unreactive with alkali metals, they actually do react, albeit slowly. To search for less reactive solvents, Li foil was immersed in a vial containing a number of dry solvents of this type in an argon-filled drybox. The reactions took many days to months to proceed to completion, with the non-cyclic amide solvents reacting the quickest, and the cyclic CHO solvents reacting the slowest. Years later, crystals were isolated from reactions with *N*,*N*-di­methyl­acetamide [DMAC, Me_2_NC(=O)Me] and tetra­methyl urea [TMU, Me_2_N(C=O)NMe_2_]. The DMAC formed a Li salt of *N*,*N*-di­methyl­acetoacetamide (*I*), possibly from deprotonation of a methyl group on one DMAC mol­ecule, which then undergoes a nucleophilic attack on a second DMAC mol­ecule. However, formation of the *N*,*N*-di­methyl­acetoacetamide ligand has been reported during solution of cellulose in DMAC containing LiCl (Potthast, *et al.*, 2003 ▸). The reaction with TMU appears to have cleaved a Me_2_N group, followed by coupling of two Me_2_N(C=O) radicals, and protonation, producing the hexa­meric Li salt of Me_2_NCH(OH)C(=O)NMe_2_. Such a coupling has been observed before from a reaction between potassium *t*-butoxide and di­methyl­formamide (DMF) (Barham *et al.*, 2016 ▸), and from a reduction using rubidium amalgam in DMF (Boeddinghaus *et al.*, 2012 ▸). While *N*,*N*-di­methyl­acetoacetamide is commercially available and has been used as a standard β-diketonate ligand, for which the V, Ti and Pd complexes have been structurally characterized (Crans *et al.*, 2001 ▸, CSD refcode ICITUJ; Gornshtein *et al.*, 2007 ▸, XEVNOC; Kiers *et al.*, 1992 ▸, JUFJID), and its Li salt has been prepared before and characterized by infrared spectroscopy (Coll, 1985 ▸), assembly of this ligand from coupling two DMAC moieties is relatively unusual, with very few references in the literature.
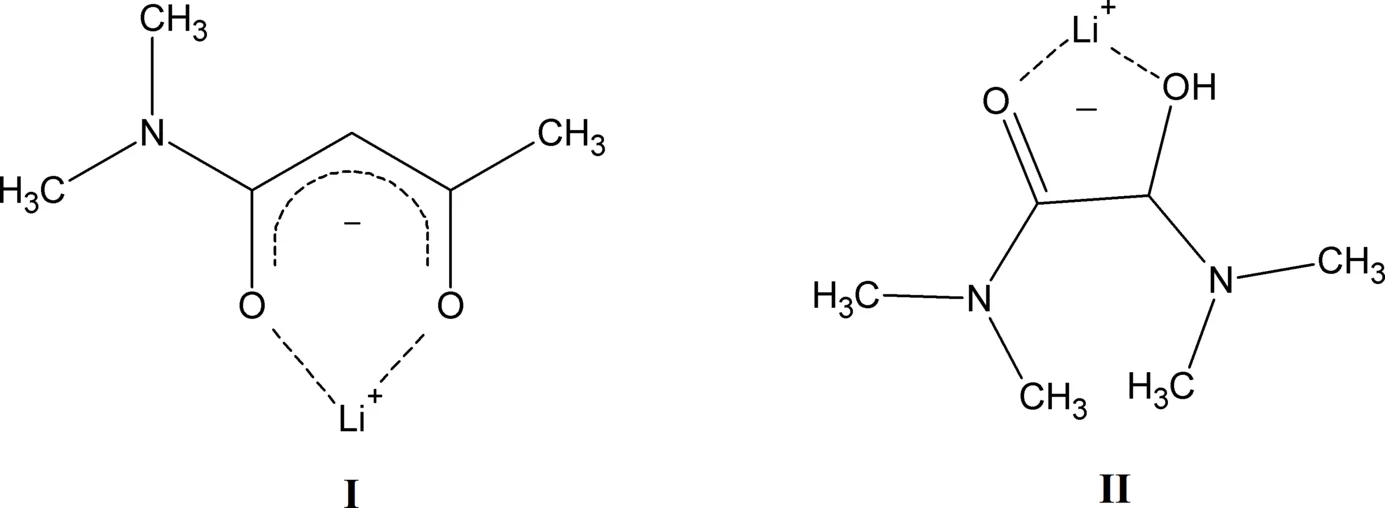


## 2. Structural commentary

**Figure 1:**
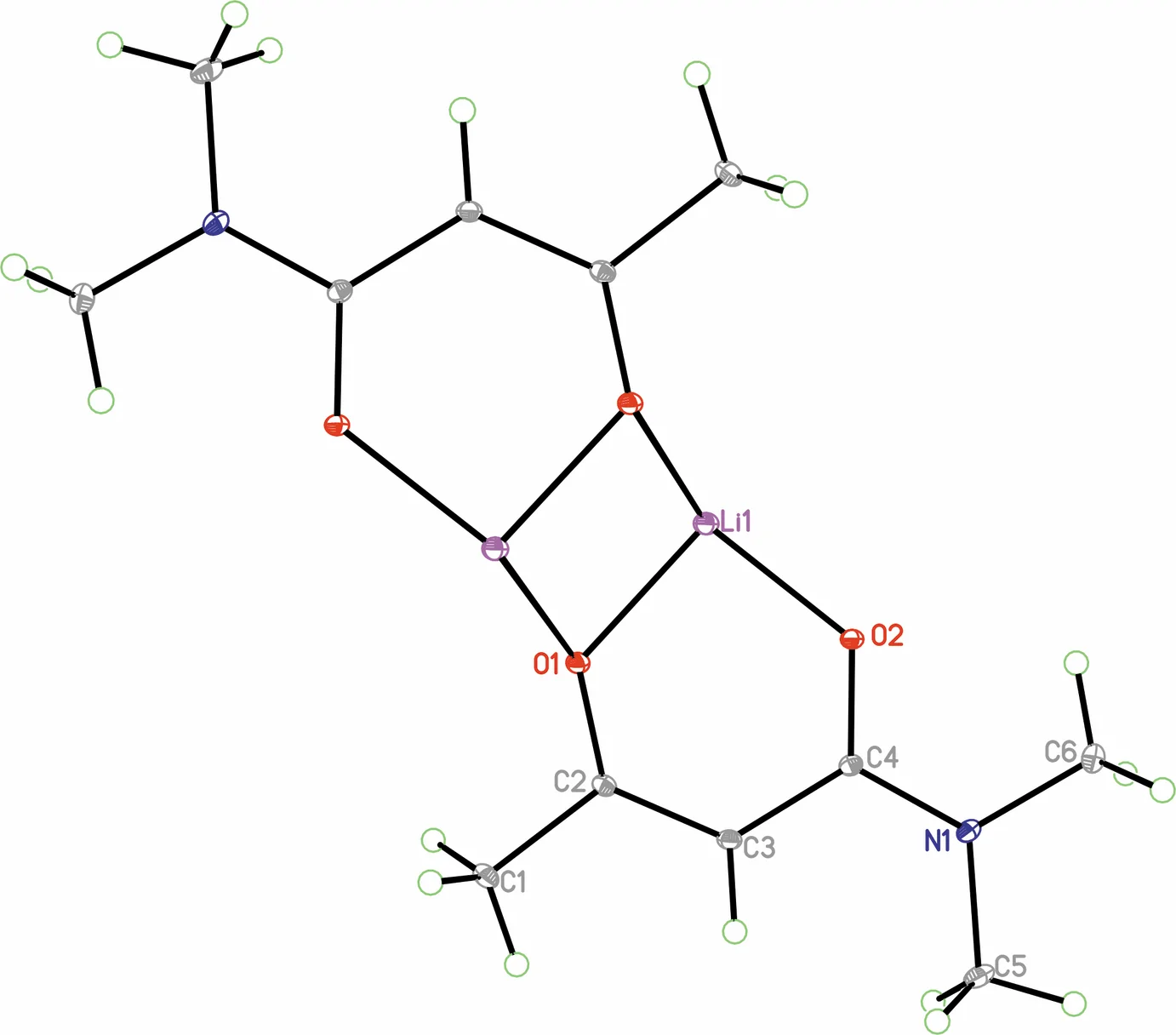
The dimer of the mol­ecular unit of **I**, showing the Li_2_O_2_ ring. Unlabeled atoms are generated by the symmetry operation 1 − *x*, −*y*, −*z*. Atomic displacement parameters are at the 30% probability level.

**Figure 2:**
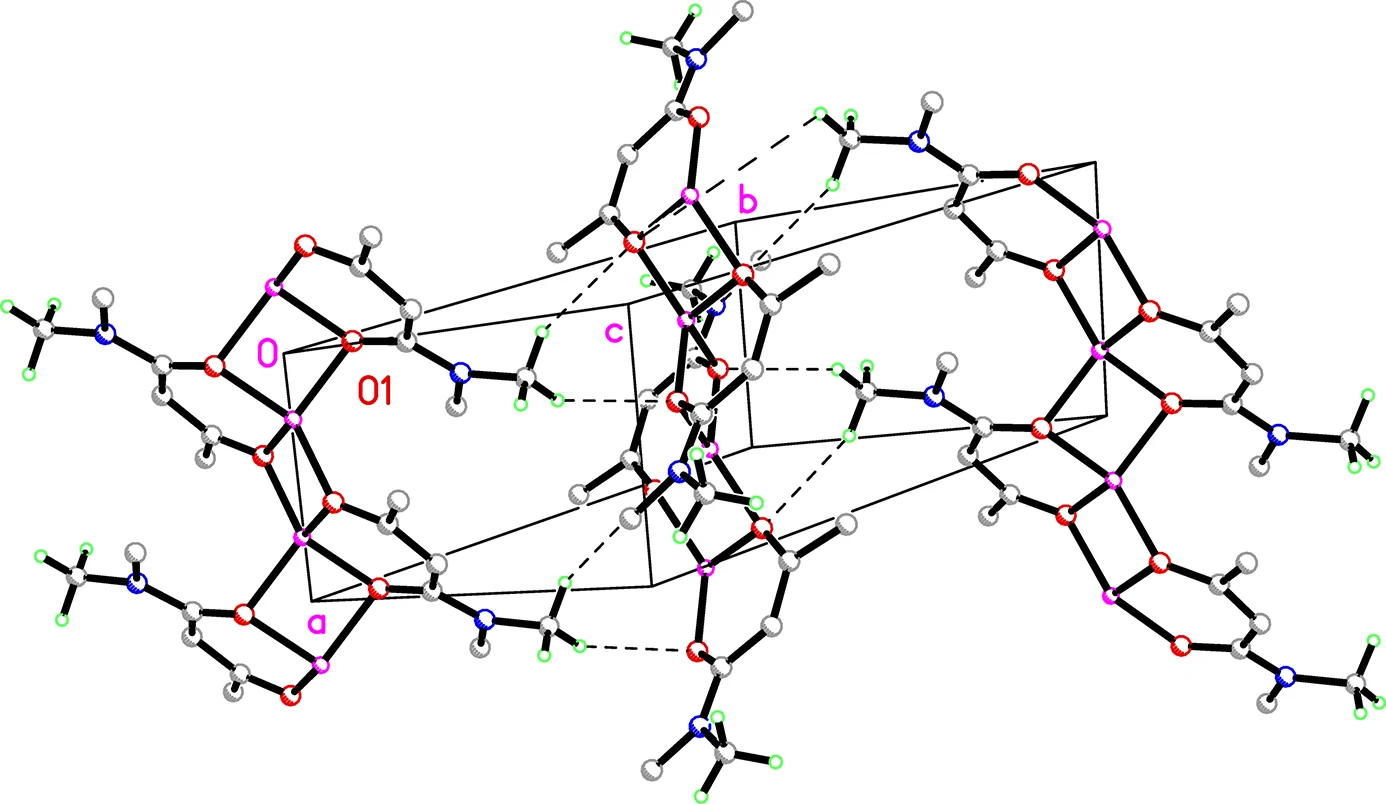
A view of **I** showing the fused Li_2_O_2_ ring chain, overlaid with the unit cell, along with the weak C—H⋯O inter­actions that support the chain.

Compound **I** crystallizes in the space group *P*2_1_/*n*. Its *N*,*N*-di­methyl­acetoacetamidate ligand coordinates as a typical β-diketonate, which is chelated to the Li^+^ ion. Selected geometrical parameters are given in Table 1 ▸. Both C=O distances of 1.2731 (10) and 1.2882 (10) Å are ordinary for mol­ecules of this type. The mol­ecules assemble into a chain of fused four-membered Li_2_O_2_ rings that zigzag along the *a*-axis direction, and the rings are slightly offset. The basic dimeric unit is shown in Fig. 1 ▸, and the assemblage in a chain is shown in Fig. 2 ▸. A plane drawn through Li1/O1/Li1^i^/O1^i^ makes an angle of 66.71 (8)° with a plane drawn through Li1/O2/Li1^ii^/O2^ii^ [symmetry codes: (i) 1 − *x*,−*y*, −*z*; (ii) 2 − *x*,−*y*, −*z*] on the adjacent Li_2_O_2_ ring along this zigzagging chain. Bond distances in the Li chelate are normal, as expected for an unstrained chelate of this type, and the Li-O rings with bond lengths of 1.9045 (17)–1.9626 (18) Å are normal as well.

**Figure 3:**
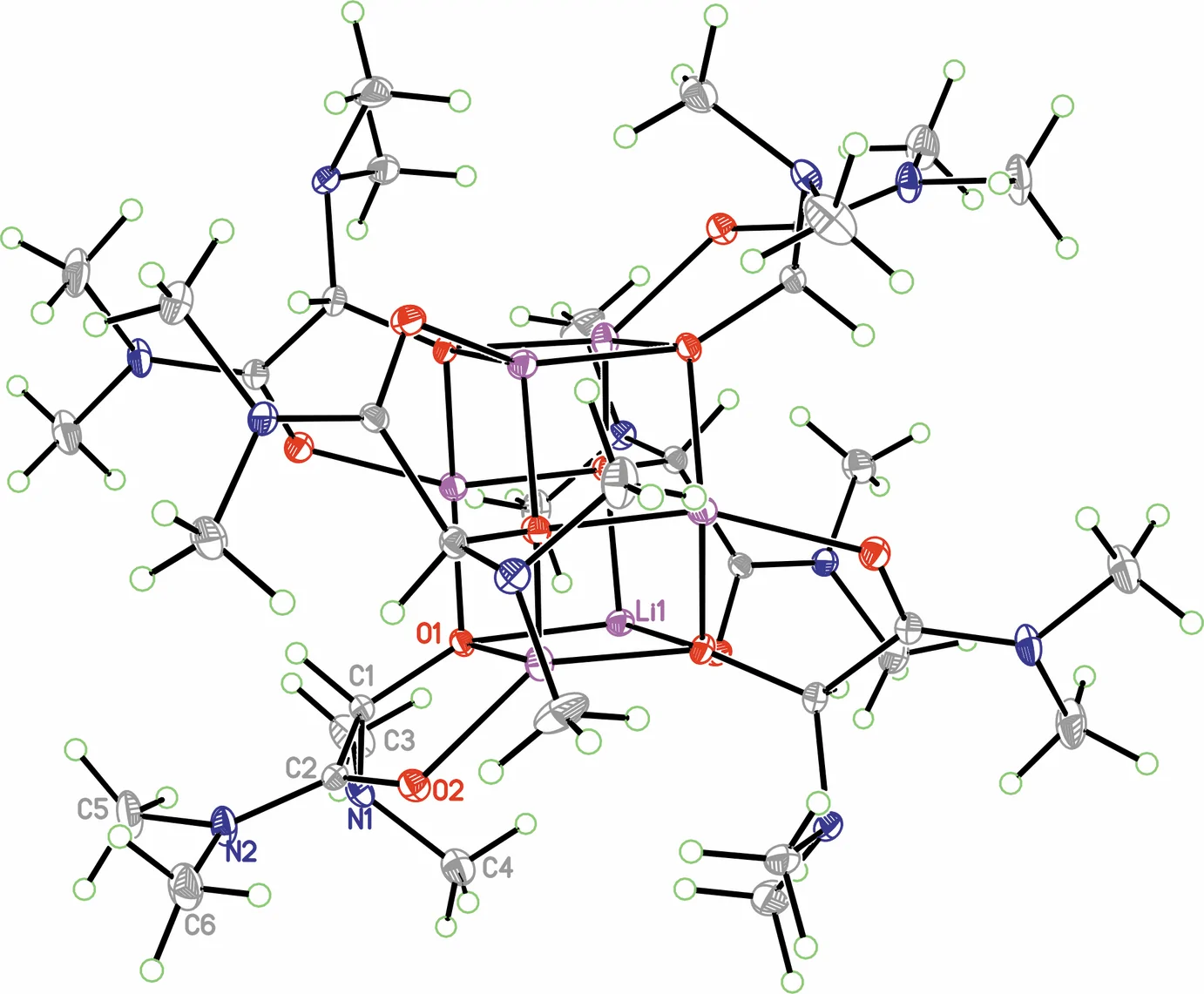
A structural diagram of **II** showing a side view of the Li_6_O_6_ cage and the bonding of the keto O atoms O2 to the cage. Unlabeled atoms are generated by the symmetry operations: −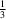 + *y*, 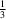 − *x* + *y*, 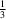 − *z*; 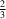 + *x* − *y*, 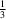 + *x*, 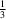 − *z*. Atomic displacement parameters are at the 30% probability level.

In 2015 the parameter τ4′ was developed to distinguish whether the geometry of the coordination center tends towards square planar or tetra­hedral (Okuniewski *et al.*, 2015 ▸). When τ4′ is close to 0 the geometry is similar to square planar, while if τ4′ is close to 1 then the geometry is similar to tetra­hedral. In this structure, the value τ4′ was found to be 0.714, which indicates a distorted tetra­hedral geometry. A search of the Cambridge Structural Database (version 6.00, update August 2025; Groom *et al.*, 2016 ▸) for similar compounds containing a five-membered chelate ring with an O–C–C–O backbone with at least one C—O bond yielded seven hits (FAQFEK, Chen *et al.*, 2009 ▸; IDOXIL, Al-Khafaji *et al.*, 2017 ▸; KIJZUZ01, Budanow *et al.*, 2010 ▸; NEFNAQ, Kingsbury *et al.*, 2017 ▸; OZUCUI, Sazama & Betley, 2011 ▸; UCUSUK, Vuković *et al.*, 2021 ▸; and VAYCUV, Wan *et al.*, 2012 ▸.). In these structures the τ4′ value was found to be 0.930, 0.760–0.826 (four different Li sites), 0.675 and 0.784 (two different Li sites),

Compound **II** crystallizes in the trigonal space group *R*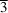. Selected geometrical parameters are given in Table 2 ▸. The core of **II** is a barrel-shaped hexa­mer consisting of two Li_3_O_3_ rings in a slightly puckered chair conformation, facially bonded together around a threefold rotation axis, (Fig. 3 ▸), which is a fairly common structural motif for Li alkoxides. For example, lithium-*t*-butoxide (Nekola *et al.*, 2002 ▸, ZZZJZU01) has a similar motif without the high crystal symmetry, and a lithium siloxide (Jones *et al.*, 1992 ▸, JOTROZ) has the threefold symmetry as well. However, this particular ligand is unusual and has been structurally characterized only once previously (Boeddinghaus *et al.*, 2012 ▸, TEXHUB), where it is coordinated to rubidium. The C1—O1 distance of 1.379 (3) Å, and the C2—O2 distance of 1.242 (3) Å are consistent with a C—O single bond and double bond, respectively, and are very similar to the corresponding distances in TEXHUB [1.346 (6) Å and 1.221 (7) Å]. Also similar are the C—N bond distances between the C=O carbon atom and its attached NMe_2_ group [C2—N2 = 1.348 (4) Å *versus* 1.345 (8) Å in TEXHUB], and the C—O (alkoxide) carbon atom and its attached NMe_2_ group [C1—N1 = 1.466 (4) Å, *versus* 1.520 (7) Å in TEXHUB], which shows some double-bond character exists between the NMe_2_ group and the carbonyl group. This results in different conformations for the two NMe_2_ groups with the environment of N1 being pyramidal while the environment for N2 being trigonal planar [C1, C2 O2, N2, C5 and C6 are almost coplanar with an r.m.s. deviation of only 0.030 (2) Å]. The O—C—C bond angles in both compounds are also almost identical [O1—C1—C2 = 110.6 (2)° and O2—C2—C1 =120.0 (3)° *versus* 110.3 (4) and 118.2 (5) ° in TEXHUB].

The τ4′ value for the coordination environment of Li in **II** is 0.760, indicating a distorted tetra­hedral conformation. A search of the Cambridge Structural Database for related Li structures containing a β-diketone ligand with both C and N substituents gave zero hits. However there are 46 structures where both substituents are carbon atoms. A typical example is BOWMAE (Petrus *et al.*, 2020 ▸) where the τ4′ value was found to be 0.772, a value similar to that in **II**.

## 3. Supra­molecular features

In the extended structure of **I**, as indicated above, the mol­ecules assemble into a chain of fused four-membered Li_2_O_2_ rings that zigzag along the *a*-axis direction. These chains are supported on both sides by inter­actions involving C5. This is a remarkable case of methyl group involved into the two types of common inter­actions simultaneously: C5 is donor of a weak C—H⋯O bond to O1 [H5*C*⋯O1(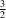 − *x*, 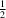 + *y*, 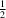 − *z*) = 2.81 Å], with a C—O distance of 3.5908 (12) Å as well as being involved in a typical tetrel bond (Varadwaj *et al.*, 2023 ▸) [C3—H3⋯O2(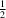 + *x*, 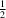 − *y*, 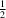 + *z*)] with a very short C—O distance of 3.0815 (12) Å as shown in Fig. 2 ▸

**Figure 4:**
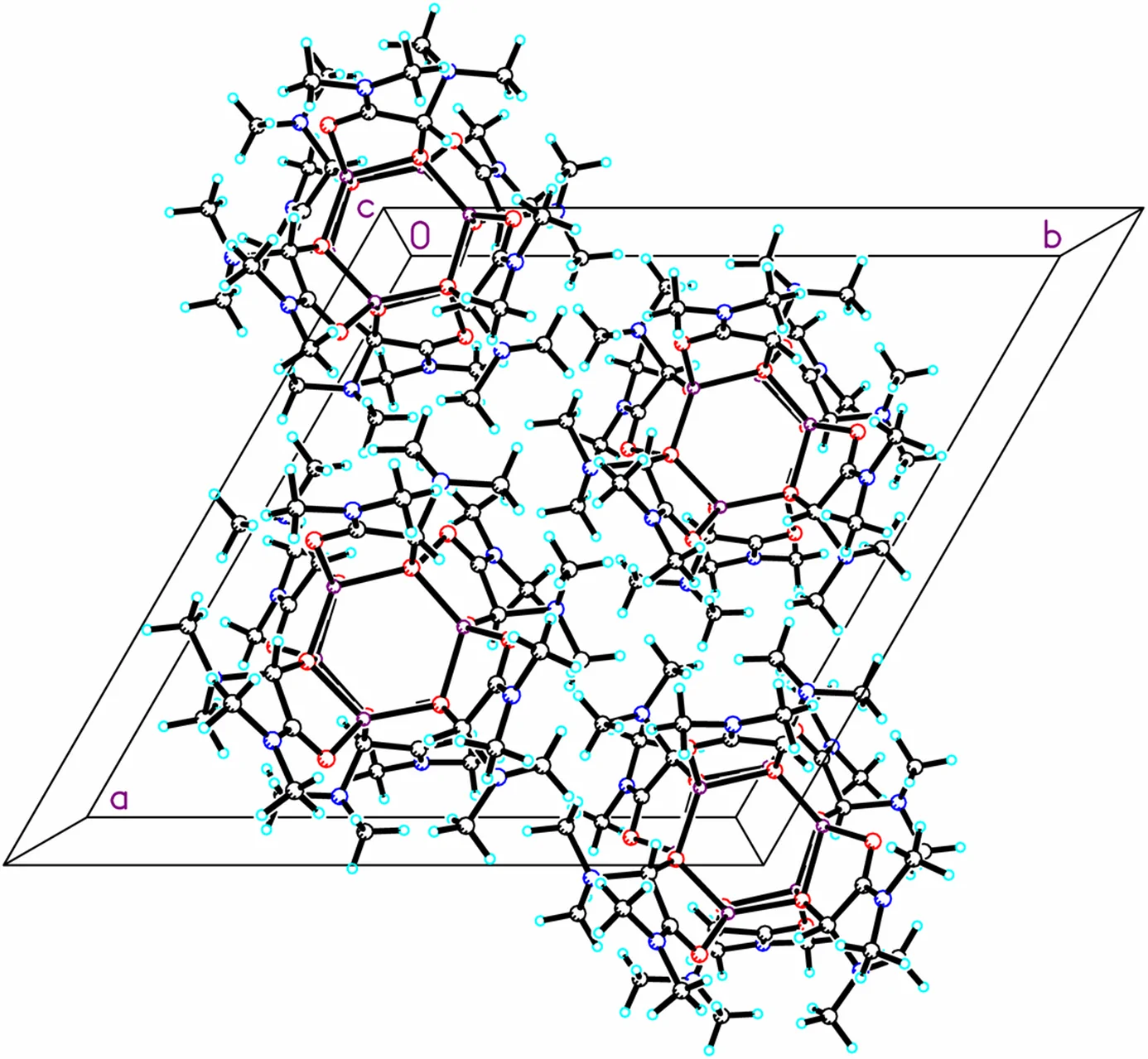
A packing diagram of **II** down the *c* axis showing the threefold symmetry.

There is weak hydrogen bonding in **II** between C—H groups and O and N donors. Both C5—H5*B*⋯N1 and C1—H1⋯O2 are notable, and help to consolidate overall structure (Table 3 ▸, Fig. 4 ▸). In the structure, the orientation of the CH_3_ methyl group involving C5 is such that there is a weak intra­molecular C5—H5*B*⋯N1 hydrogen bond.

## 4. Database survey

A search of the CSD database (version 6.00, update August 2025; Groom *et al.*, 2016 ▸) found 35 instances of a barrel-shaped Li_6_O_6_ cluster similar to that in **II**, mostly in lithium alkoxides and siloxides, with additional structures where that motif is a subunit of a larger cluster. The great structural flexibility of Li–O clusters results in many other structural patterns, for example, stacked cubes (Goldfuss *et al.*, 1997 ▸, NOXWEC) or prism structures (Dell’Aera *et al.* 2024 ▸, UHUWUT01). While the polymeric fused Li_2_O_2_ chains of **I** are less common, there are still plenty of examples of this general motif, particularly with β-diketonate salts such as the lithium salt of 1-eth­oxy-3-di­fluoro­methyl-1,3-di­propanone, and its tri­fluoro­methyl analog (Slepukhin *et al.*, 2009 ▸ EJUZIT, EJUZOZ), and the phenyl di­fluoro­methyl analog as well (Karpenko *et al.*, 2005 ▸, REGQAW).

## 5. Synthesis and crystallization

A piece of Li foil of about 0.1 g was added to scintillation vials containing ∼10 mL of dry tetra­methyl urea (TMU), *N*,*N*-di­methyl­acetamide (DMAC), γ-valerolactone, and *N*,*N*-di­methyl­imidazolidinone (DMIZ) inside an argon-filled drybox. Within three days, the Li in the TMU dissolved to an orange solution, and the piece in DMAC reacted to form a yellow solution and a white precipitate, but the ones in DMIZ and γ-valerolactone did not fully react until three months had passed. Suitable crystals were isolated several years later from the DMAC and TMU mixtures. The solids isolated from the other two vials were not suitable for x-ray diffraction.

## 6. Refinement

Crystal data, data collection and structure refinement details are summarized in Table 4 ▸. For both **I** and **II**, all H atoms were located in difference maps and treated as riding in geometrically idealized positions with C—H distances of 0.98 Å for the methyl groups. For **II** there was unresolved solvent peaks along the threefold axis. This was treated by using the SQUEEZE routine in *PLATON* (Spek, 2015 ▸)

## Supplementary Material

Crystal structure: contains datablock(s) I, II. DOI: 10.1107/S2056989026009989/vu2021sup1.cif

Structure factors: contains datablock(s) I. DOI: 10.1107/S2056989026009989/vu2021Isup2.hkl

Structure factors: contains datablock(s) II. DOI: 10.1107/S2056989026009989/vu2021IIsup3.hkl

CCDC references: 2590805, 2590806

Additional supporting information:  crystallographic information; 3D view; checkCIF report

## Figures and Tables

**Table 1:** Selected geometric parameters (Å, °) for **I**[Chem scheme1]

O1—Li1	1.9071 (17)	O2—Li1^ii^	1.9626 (18)
O1—Li1^i^	1.9376 (18)	Li1—Li1^i^	2.594 (3)
O2—Li1	1.9045 (17)	Li1—Li1^ii^	2.690 (3)
			
O2—Li1—O1	95.19 (8)	O2—Li1—O2^ii^	91.86 (7)
O2—Li1—O1^i^	139.00 (10)	O1—Li1—O2^ii^	124.75 (9)
O1—Li1—O1^i^	95.13 (7)	O1^i^—Li1—O2^ii^	113.88 (8)

Symmetry codes: (i) 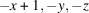; (ii) 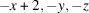.

**Table 2:** Selected geometric parameters (Å, °) for **II**[Chem scheme1]

O1—Li1	1.923 (5)	O1—Li1^ii^	1.974 (5)
O1—Li1^i^	1.940 (5)	O2—Li1^ii^	1.944 (5)
			
O1—Li1—O1^ii^	99.6 (2)	O1—Li1—O1^i^	98.4 (2)
O1—Li1—O2^i^	141.2 (3)	O1^ii^—Li1—O1^i^	120.4 (3)
O1^ii^—Li1—O2^i^	111.4 (2)	O2^i^—Li1—O1^i^	85.8 (2)

Symmetry codes: (i) 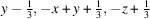; (ii) 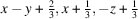.

**Table 3:** Hydrogen-bond geometry (Å, °) for **II**[Chem scheme1]

*D*—H⋯*A*	*D*—H	H⋯*A*	*D*⋯*A*	*D*—H⋯*A*
C1—H1⋯O2^iii^	1.00	2.59	3.322 (4)	130
C5—H5*B*⋯N1	0.98	2.34	3.059 (4)	130

Symmetry code: (iii) 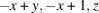.

**Table 4:** Experimental details

	**I**	**II**
Crystal data		
Chemical formula	[Li(C_6_H_10_NO_2_)]	[Li_6_(C_6_H_13_N_2_O_2_)_6_]
*M*_r_	135.09	912.74
Crystal system, space group	Monoclinic, *P*2_1_/*n*	Trigonal, *R* 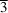
Temperature (K)	100	116
*a*, *b*, *c* (Å)	5.2478 (2), 12.3868 (5), 10.9921 (5)	18.156 (4), 18.156 (4), 13.698 (4)
α, β, γ (°)	90, 103.461 (2), 90	90, 90, 120
*V* (Å^3^)	694.90 (5)	3911 (2)
*Z*	4	3
Radiation type	Mo *K*α	Mo *K*α
μ (mm^−1^)	0.09	0.09
Crystal size (mm)	0.26 × 0.18 × 0.12	0.15 × 0.12 × 0.05

Data collection		
Diffractometer	Bruker APEXII CCD	Bruker APEXII CCD
Absorption correction	Multi-scan (*SADABS*; Krause *et al.*, 2015 ▸)	Multi-scan (*SADABS*; Krause *et al.*, 2015 ▸)
*T*_min_, *T*_max_	0.635, 0.743	0.393, 0.745
No. of measured, independent and observed [*I* > 2σ(*I*)] reflections	17985, 3219, 2619	5186, 1589, 915
*R*_int_	0.050	0.094
(sin θ/λ)_max_ (Å^−1^)	0.844	0.602

Refinement		
*R*[*F*^2^ > 2σ(*F*^2^)], *wR*(*F*^2^), *S*	0.048, 0.126, 1.06	0.064, 0.163, 1.01
No. of reflections	3219	1589
No. of parameters	96	104
H-atom treatment	H-atom parameters constrained	H-atom parameters constrained
Δρ_max_, Δρ_min_ (e Å^−3^)	0.57, −0.23	0.43, −0.22

Computer programs: *APEX2* (Bruker, 2005 ▸), *SAINT* (Bruker, 2002 ▸), *SHELXT* (Sheldrick 2015*a* ▸), *SHELXL2019/2* (Sheldrick, 2015*b* ▸) and *SHELXTL* (Sheldrick, 2008 ▸).

## supplementary crystallographic information

### catena-Poly[(µ3-N,N-dimethylacetoacetamidato)lithium(I)] (I) Crystal data

**Table:**

[Li(C_6_H_10_NO_2_)]	*F*(000) = 288
*M**_r_* = 135.09	*D*_x_ = 1.291 Mg m^−^^3^
Monoclinic, *P*2_1_/*n*	Mo *K*α radiation, λ = 0.71073 Å
*a* = 5.2478 (2) Å	Cell parameters from 6211 reflections
*b* = 12.3868 (5) Å	θ = 3.3–30.6°
*c* = 10.9921 (5) Å	µ = 0.09 mm^−^^1^
β = 103.461 (2)°	*T* = 100 K
*V* = 694.90 (5) Å^3^	Prism, colourless
*Z* = 4	0.26 × 0.18 × 0.12 mm

### catena-Poly[(µ3-N,N-dimethylacetoacetamidato)lithium(I)] (I) Data collection

**Table:**

Bruker APEXII CCD diffractometer	2619 reflections with *I* > 2σ(*I*)
φ and ω scans	*R*_int_ = 0.050
Absorption correction: multi-scan (SADABS; Krause *et al.*, 2015)	θ_max_ = 36.8°, θ_min_ = 3.3°
*T*_min_ = 0.635, *T*_max_ = 0.743	*h* = −8→8
17985 measured reflections	*k* = −19→19
3219 independent reflections	*l* = −18→17

### catena-Poly[(µ3-N,N-dimethylacetoacetamidato)lithium(I)] (I) Refinement

**Table:**

Refinement on *F*^2^	Primary atom site location: dual
Least-squares matrix: full	Secondary atom site location: difference Fourier map
*R*[*F*^2^ > 2σ(*F*^2^)] = 0.048	Hydrogen site location: inferred from neighbouring sites
*wR*(*F*^2^) = 0.126	H-atom parameters constrained
*S* = 1.06	*w* = 1/[σ^2^(*F*_o_^2^) + (0.0607*P*)^2^ + 0.1969*P*] where *P* = (*F*_o_^2^ + 2*F*_c_^2^)/3
3219 reflections	(Δ/σ)_max_ < 0.001
96 parameters	Δρ_max_ = 0.57 e Å^−^^3^
0 restraints	Δρ_min_ = −0.23 e Å^−^^3^

### catena-Poly[(µ3-N,N-dimethylacetoacetamidato)lithium(I)] (I) Special details

**Table:**

Geometry. All esds (except the esd in the dihedral angle between two l.s. planes) are estimated using the full covariance matrix. The cell esds are taken into account individually in the estimation of esds in distances, angles and torsion angles; correlations between esds in cell parameters are only used when they are defined by crystal symmetry. An approximate (isotropic) treatment of cell esds is used for estimating esds involving l.s. planes.

### catena-Poly[(µ3-N,N-dimethylacetoacetamidato)lithium(I)] (I) Fractional atomic coordinates and isotropic or equivalent isotropic displacement parameters (Å^2^)

**Table:**

	*x*	*y*	*z*	*U*_iso_*/*U*_eq_	
O1	0.59962 (12)	−0.01383 (5)	0.13056 (6)	0.01357 (13)	
O2	1.01783 (12)	0.09654 (5)	0.06579 (6)	0.01264 (12)	
N1	1.23735 (15)	0.21292 (6)	0.21082 (7)	0.01497 (14)	
C1	0.61051 (18)	−0.02321 (8)	0.34787 (8)	0.01594 (16)	
H1A	0.712848	0.006620	0.426777	0.024*	
H1B	0.622423	−0.102169	0.350360	0.024*	
H1C	0.426848	−0.001393	0.335987	0.024*	
C2	0.71778 (16)	0.01900 (7)	0.24042 (7)	0.01192 (14)	
C3	0.92905 (16)	0.08955 (7)	0.26828 (7)	0.01299 (14)	
H3	0.991510	0.110908	0.353001	0.016*	
C4	1.05980 (15)	0.13250 (6)	0.17718 (7)	0.01154 (14)	
C5	1.3038 (2)	0.25888 (8)	0.33664 (9)	0.02047 (18)	
H5A	1.428371	0.318298	0.339481	0.031*	
H5B	1.383134	0.203015	0.396732	0.031*	
H5C	1.144541	0.286157	0.358115	0.031*	
C6	1.38164 (17)	0.25529 (7)	0.12269 (9)	0.01694 (16)	
H6A	1.361515	0.333922	0.117195	0.025*	
H6B	1.312649	0.223310	0.040036	0.025*	
H6C	1.567660	0.237110	0.151656	0.025*	
Li1	0.7387 (3)	0.00402 (14)	−0.01375 (15)	0.0151 (3)	

### catena-Poly[(µ3-N,N-dimethylacetoacetamidato)lithium(I)] (I) Atomic displacement parameters (Å^2^)

**Table:**

	*U*^11^	*U*^22^	*U*^33^	*U*^12^	*U*^13^	*U*^23^
O1	0.0121 (3)	0.0182 (3)	0.0102 (2)	−0.0017 (2)	0.00199 (19)	−0.0012 (2)
O2	0.0130 (3)	0.0150 (3)	0.0101 (2)	−0.0012 (2)	0.00284 (18)	−0.00230 (19)
N1	0.0155 (3)	0.0144 (3)	0.0145 (3)	−0.0036 (2)	0.0025 (2)	−0.0038 (2)
C1	0.0161 (4)	0.0207 (4)	0.0118 (3)	0.0009 (3)	0.0049 (3)	0.0025 (3)
C2	0.0114 (3)	0.0142 (3)	0.0102 (3)	0.0023 (2)	0.0026 (2)	0.0006 (2)
C3	0.0133 (3)	0.0160 (3)	0.0094 (3)	−0.0003 (2)	0.0022 (2)	−0.0018 (2)
C4	0.0109 (3)	0.0116 (3)	0.0116 (3)	0.0009 (2)	0.0013 (2)	−0.0010 (2)
C5	0.0234 (4)	0.0185 (4)	0.0175 (4)	−0.0040 (3)	0.0006 (3)	−0.0072 (3)
C6	0.0151 (4)	0.0152 (4)	0.0203 (4)	−0.0023 (3)	0.0038 (3)	0.0011 (3)
Li1	0.0140 (7)	0.0192 (7)	0.0122 (6)	−0.0015 (5)	0.0031 (5)	−0.0023 (5)

### catena-Poly[(µ3-N,N-dimethylacetoacetamidato)lithium(I)] (I) Geometric parameters (Å, º)

**Table:**

O1—C2	1.2882 (10)	C1—H1C	0.9800
O1—Li1	1.9071 (17)	C2—C3	1.3888 (12)
O1—Li1^i^	1.9376 (18)	C3—C4	1.4410 (11)
O2—C4	1.2731 (10)	C3—H3	0.9500
O2—Li1	1.9045 (17)	C5—H5A	0.9800
O2—Li1^ii^	1.9626 (18)	C5—H5B	0.9800
N1—C4	1.3553 (11)	C5—H5C	0.9800
N1—C6	1.4590 (12)	C6—H6A	0.9800
N1—C5	1.4608 (12)	C6—H6B	0.9800
C1—C2	1.5149 (11)	C6—H6C	0.9800
C1—H1A	0.9800	Li1—Li1^i^	2.594 (3)
C1—H1B	0.9800	Li1—Li1^ii^	2.690 (3)
			
C2—O1—Li1	123.31 (7)	H5A—C5—H5B	109.5
C2—O1—Li1^i^	140.20 (7)	N1—C5—H5C	109.5
Li1—O1—Li1^i^	84.87 (7)	H5A—C5—H5C	109.5
C4—O2—Li1	126.47 (7)	H5B—C5—H5C	109.5
C4—O2—Li1^ii^	121.59 (7)	N1—C6—H6A	109.5
Li1—O2—Li1^ii^	88.14 (7)	N1—C6—H6B	109.5
C4—N1—C6	120.89 (7)	H6A—C6—H6B	109.5
C4—N1—C5	122.83 (8)	N1—C6—H6C	109.5
C6—N1—C5	116.23 (7)	H6A—C6—H6C	109.5
C2—C1—H1A	109.5	H6B—C6—H6C	109.5
C2—C1—H1B	109.5	O2—Li1—O1	95.19 (8)
H1A—C1—H1B	109.5	O2—Li1—O1^i^	139.00 (10)
C2—C1—H1C	109.5	O1—Li1—O1^i^	95.13 (7)
H1A—C1—H1C	109.5	O2—Li1—O2^ii^	91.86 (7)
H1B—C1—H1C	109.5	O1—Li1—O2^ii^	124.75 (9)
O1—C2—C3	126.21 (7)	O1^i^—Li1—O2^ii^	113.88 (8)
O1—C2—C1	115.90 (7)	O2—Li1—Li1^i^	129.06 (11)
C3—C2—C1	117.89 (7)	O1—Li1—Li1^i^	48.06 (6)
C2—C3—C4	124.33 (7)	O1^i^—Li1—Li1^i^	47.07 (6)
C2—C3—H3	117.8	O2^ii^—Li1—Li1^i^	136.17 (11)
C4—C3—H3	117.8	O2—Li1—Li1^ii^	46.82 (5)
O2—C4—N1	118.27 (7)	O1—Li1—Li1^ii^	118.67 (10)
O2—C4—C3	122.38 (7)	O1^i^—Li1—Li1^ii^	146.07 (11)
N1—C4—C3	119.35 (7)	O2^ii^—Li1—Li1^ii^	45.04 (5)
N1—C5—H5A	109.5	Li1^i^—Li1—Li1^ii^	166.50 (14)
N1—C5—H5B	109.5		
			
Li1—O1—C2—C3	13.93 (13)	Li1—O2—C4—C3	13.19 (13)
Li1^i^—O1—C2—C3	−114.50 (12)	Li1^ii^—O2—C4—C3	−100.84 (10)
Li1—O1—C2—C1	−166.94 (8)	C6—N1—C4—O2	−1.56 (12)
Li1^i^—O1—C2—C1	64.63 (14)	C5—N1—C4—O2	−178.94 (8)
O1—C2—C3—C4	−3.85 (14)	C6—N1—C4—C3	177.40 (8)
C1—C2—C3—C4	177.04 (8)	C5—N1—C4—C3	0.02 (12)
Li1—O2—C4—N1	−167.88 (8)	C2—C3—C4—O2	−10.97 (13)
Li1^ii^—O2—C4—N1	78.09 (11)	C2—C3—C4—N1	170.12 (8)

Symmetry codes: (i) −*x*+1, −*y*, −*z*; (ii) −*x*+2, −*y*, −*z*.

### Hexakis[µ3-1,2-bis(dimethylamino)-2-oxoethan-1-olato]hexalithium(I) (II) Crystal data

**Table:**

[Li_6_(C_6_H_13_N_2_O_2_)_6_]	*D*_x_ = 1.163 Mg m^−^^3^
*M**_r_* = 912.74	Mo *K*α radiation, λ = 0.71073 Å
Trigonal, *R*3	Cell parameters from 894 reflections
*a* = 18.156 (4) Å	θ = 3.0–24.8°
*c* = 13.698 (4) Å	µ = 0.09 mm^−^^1^
*V* = 3911 (2) Å^3^	*T* = 116 K
*Z* = 3	Prism, colorless
*F*(000) = 1476	0.15 × 0.12 × 0.05 mm

### Hexakis[µ3-1,2-bis(dimethylamino)-2-oxoethan-1-olato]hexalithium(I) (II) Data collection

**Table:**

Bruker APEXII CCD diffractometer	915 reflections with *I* > 2σ(*I*)
φ and ω scans	*R*_int_ = 0.094
Absorption correction: multi-scan (SADABS; Krause *et al.*, 2015)	θ_max_ = 25.3°, θ_min_ = 3.0°
*T*_min_ = 0.393, *T*_max_ = 0.745	*h* = −21→18
5186 measured reflections	*k* = −19→21
1589 independent reflections	*l* = −13→16

### Hexakis[µ3-1,2-bis(dimethylamino)-2-oxoethan-1-olato]hexalithium(I) (II) Refinement

**Table:**

Refinement on *F*^2^	Primary atom site location: dual
Least-squares matrix: full	Secondary atom site location: difference Fourier map
*R*[*F*^2^ > 2σ(*F*^2^)] = 0.064	Hydrogen site location: inferred from neighbouring sites
*wR*(*F*^2^) = 0.163	H-atom parameters constrained
*S* = 1.01	*w* = 1/[σ^2^(*F*_o_^2^) + (0.0741*P*)^2^] where *P* = (*F*_o_^2^ + 2*F*_c_^2^)/3
1589 reflections	(Δ/σ)_max_ < 0.001
104 parameters	Δρ_max_ = 0.43 e Å^−^^3^
0 restraints	Δρ_min_ = −0.22 e Å^−^^3^

### Hexakis[µ3-1,2-bis(dimethylamino)-2-oxoethan-1-olato]hexalithium(I) (II) Special details

**Table:**

Geometry. All esds (except the esd in the dihedral angle between two l.s. planes) are estimated using the full covariance matrix. The cell esds are taken into account individually in the estimation of esds in distances, angles and torsion angles; correlations between esds in cell parameters are only used when they are defined by crystal symmetry. An approximate (isotropic) treatment of cell esds is used for estimating esds involving l.s. planes.

### Hexakis[µ3-1,2-bis(dimethylamino)-2-oxoethan-1-olato]hexalithium(I) (II) Fractional atomic coordinates and isotropic or equivalent isotropic displacement parameters (Å^2^)

**Table:**

	*x*	*y*	*z*	*U*_iso_*/*U*_eq_	
O1	0.35939 (12)	0.57409 (12)	0.24712 (13)	0.0223 (5)	
O2	0.50447 (13)	0.67719 (13)	0.33958 (15)	0.0282 (6)	
N1	0.38352 (16)	0.46658 (15)	0.32016 (17)	0.0276 (7)	
N2	0.46926 (16)	0.59789 (17)	0.47645 (17)	0.0333 (7)	
C1	0.37433 (18)	0.54192 (18)	0.3312 (2)	0.0232 (7)	
H1	0.325206	0.526187	0.376097	0.028*	
C2	0.45485 (18)	0.61048 (19)	0.3831 (2)	0.0227 (7)	
C3	0.3028 (2)	0.3907 (2)	0.2969 (3)	0.0533 (11)	
H3A	0.309407	0.340456	0.301229	0.080*	
H3B	0.285549	0.395578	0.230512	0.080*	
H3C	0.259255	0.385195	0.343320	0.080*	
C4	0.4479 (2)	0.4799 (2)	0.2475 (2)	0.0403 (9)	
H4A	0.459547	0.432748	0.250370	0.061*	
H4B	0.500227	0.533471	0.261159	0.061*	
H4C	0.427018	0.482353	0.182310	0.061*	
C5	0.4119 (2)	0.5224 (2)	0.5341 (2)	0.0505 (11)	
H5A	0.445502	0.506740	0.576227	0.076*	
H5B	0.376906	0.475082	0.489987	0.076*	
H5C	0.374993	0.535034	0.574409	0.076*	
C6	0.5401 (2)	0.6652 (2)	0.5305 (2)	0.0484 (10)	
H6A	0.573690	0.642522	0.560430	0.073*	
H6B	0.518138	0.686848	0.581628	0.073*	
H6C	0.576184	0.711568	0.485828	0.073*	
Li1	0.3600 (3)	0.5773 (3)	0.1068 (3)	0.0268 (12)	

### Hexakis[µ3-1,2-bis(dimethylamino)-2-oxoethan-1-olato]hexalithium(I) (II) Atomic displacement parameters (Å^2^)

**Table:**

	*U*^11^	*U*^22^	*U*^33^	*U*^12^	*U*^13^	*U*^23^
O1	0.0223 (12)	0.0237 (11)	0.0194 (11)	0.0106 (9)	−0.0005 (9)	0.0045 (9)
O2	0.0244 (12)	0.0263 (12)	0.0294 (12)	0.0092 (10)	−0.0032 (10)	0.0013 (10)
N1	0.0329 (15)	0.0223 (14)	0.0266 (14)	0.0130 (13)	−0.0032 (12)	0.0040 (11)
N2	0.0299 (16)	0.0381 (17)	0.0180 (13)	0.0065 (13)	−0.0027 (12)	0.0008 (12)
C1	0.0236 (17)	0.0286 (18)	0.0189 (15)	0.0142 (14)	−0.0007 (13)	0.0020 (13)
C2	0.0198 (16)	0.0278 (17)	0.0207 (16)	0.0121 (15)	0.0024 (13)	0.0012 (14)
C3	0.054 (2)	0.0229 (19)	0.066 (3)	0.0070 (18)	−0.025 (2)	−0.0015 (18)
C4	0.064 (3)	0.039 (2)	0.0335 (19)	0.037 (2)	−0.0022 (18)	−0.0027 (16)
C5	0.050 (2)	0.060 (3)	0.0223 (17)	0.013 (2)	0.0010 (17)	0.0122 (18)
C6	0.047 (2)	0.057 (3)	0.0276 (19)	0.016 (2)	−0.0081 (17)	−0.0070 (18)
Li1	0.021 (3)	0.036 (3)	0.024 (3)	0.014 (2)	−0.001 (2)	−0.001 (2)

### Hexakis[µ3-1,2-bis(dimethylamino)-2-oxoethan-1-olato]hexalithium(I) (II) Geometric parameters (Å, º)

**Table:**

O1—C1	1.379 (3)	C3—H3A	0.9800
O1—Li1	1.923 (5)	C3—H3B	0.9800
O1—Li1^i^	1.940 (5)	C3—H3C	0.9800
O1—Li1^ii^	1.974 (5)	C4—H4A	0.9800
O2—C2	1.242 (3)	C4—H4B	0.9800
O2—Li1^ii^	1.944 (5)	C4—H4C	0.9800
N1—C3	1.459 (4)	C5—H5A	0.9800
N1—C4	1.460 (4)	C5—H5B	0.9800
N1—C1	1.466 (4)	C5—H5C	0.9800
N2—C2	1.348 (4)	C6—H6A	0.9800
N2—C6	1.458 (4)	C6—H6B	0.9800
N2—C5	1.469 (4)	C6—H6C	0.9800
C1—C2	1.540 (4)	Li1—Li1^ii^	2.518 (7)
C1—Li1^ii^	2.779 (5)	Li1—Li1^i^	2.519 (7)
C1—H1	1.0000	Li1—Li1^iii^	3.310 (9)
C2—Li1^ii^	2.649 (6)	Li1—Li1^iv^	3.310 (9)
			
C1—O1—Li1	148.1 (2)	H6A—C6—H6B	109.5
C1—O1—Li1^i^	116.6 (2)	N2—C6—H6C	109.5
Li1—O1—Li1^i^	81.4 (2)	H6A—C6—H6C	109.5
C1—O1—Li1^ii^	110.7 (2)	H6B—C6—H6C	109.5
Li1—O1—Li1^ii^	80.5 (2)	O1—Li1—O1^ii^	99.6 (2)
Li1^i^—O1—Li1^ii^	115.5 (3)	O1—Li1—O2^i^	141.2 (3)
C2—O2—Li1^ii^	110.6 (2)	O1^ii^—Li1—O2^i^	111.4 (2)
C3—N1—C4	110.5 (3)	O1—Li1—O1^i^	98.4 (2)
C3—N1—C1	112.0 (2)	O1^ii^—Li1—O1^i^	120.4 (3)
C4—N1—C1	111.8 (2)	O2^i^—Li1—O1^i^	85.8 (2)
C2—N2—C6	120.3 (3)	O1—Li1—Li1^ii^	50.6 (2)
C2—N2—C5	124.1 (3)	O1^ii^—Li1—Li1^ii^	49.02 (11)
C6—N2—C5	115.2 (3)	O2^i^—Li1—Li1^ii^	153.5 (3)
O1—C1—N1	116.6 (2)	O1^i^—Li1—Li1^ii^	118.64 (17)
O1—C1—C2	110.6 (2)	O1—Li1—Li1^i^	49.6 (2)
N1—C1—C2	107.3 (2)	O1^ii^—Li1—Li1^i^	119.20 (18)
O1—C1—Li1^ii^	41.64 (16)	O2^i^—Li1—Li1^i^	123.9 (2)
N1—C1—Li1^ii^	129.9 (2)	O1^i^—Li1—Li1^i^	48.86 (11)
C2—C1—Li1^ii^	68.94 (18)	Li1^ii^—Li1—Li1^i^	82.2 (3)
O1—C1—H1	107.4	O1—Li1—C2^i^	142.9 (3)
N1—C1—H1	107.4	O1^ii^—Li1—C2^i^	117.3 (2)
C2—C1—H1	107.4	O2^i^—Li1—C2^i^	26.03 (11)
Li1^ii^—C1—H1	121.7	O1^i^—Li1—C2^i^	60.49 (15)
O2—C2—N2	120.9 (3)	Li1^ii^—Li1—C2^i^	165.2 (3)
O2—C2—C1	120.0 (3)	Li1^i^—Li1—C2^i^	104.1 (2)
N2—C2—C1	119.1 (3)	O1—Li1—C1^i^	120.8 (2)
O2—C2—Li1^ii^	43.38 (17)	O1^ii^—Li1—C1^i^	124.5 (2)
N2—C2—Li1^ii^	158.4 (2)	O2^i^—Li1—C1^i^	58.31 (15)
C1—C2—Li1^ii^	78.21 (18)	O1^i^—Li1—C1^i^	27.65 (10)
N1—C3—H3A	109.5	Li1^ii^—Li1—C1^i^	144.72 (16)
N1—C3—H3B	109.5	Li1^i^—Li1—C1^i^	73.54 (16)
H3A—C3—H3B	109.5	C2^i^—Li1—C1^i^	32.85 (10)
N1—C3—H3C	109.5	O1—Li1—Li1^iii^	91.59 (16)
H3A—C3—H3C	109.5	O1^ii^—Li1—Li1^iii^	32.56 (17)
H3B—C3—H3C	109.5	O2^i^—Li1—Li1^iii^	127.03 (16)
N1—C4—H4A	109.5	O1^i^—Li1—Li1^iii^	90.95 (15)
N1—C4—H4B	109.5	Li1^ii^—Li1—Li1^iii^	48.92 (13)
H4A—C4—H4B	109.5	Li1^i^—Li1—Li1^iii^	90.0
N1—C4—H4C	109.5	C2^i^—Li1—Li1^iii^	116.97 (12)
H4A—C4—H4C	109.5	C1^i^—Li1—Li1^iii^	105.04 (14)
H4B—C4—H4C	109.5	O1—Li1—Li1^iv^	90.98 (16)
N2—C5—H5A	109.5	O1^ii^—Li1—Li1^iv^	91.57 (16)
N2—C5—H5B	109.5	O2^i^—Li1—Li1^iv^	110.2 (2)
H5A—C5—H5B	109.5	O1^i^—Li1—Li1^iv^	31.93 (16)
N2—C5—H5C	109.5	Li1^ii^—Li1—Li1^iv^	90.0
H5A—C5—H5C	109.5	Li1^i^—Li1—Li1^iv^	48.92 (13)
H5B—C5—H5C	109.5	C2^i^—Li1—Li1^iv^	84.46 (17)
N2—C6—H6A	109.5	C1^i^—Li1—Li1^iv^	54.75 (16)
N2—C6—H6B	109.5	Li1^iii^—Li1—Li1^iv^	60.0
			
Li1—O1—C1—N1	−15.2 (6)	Li1^ii^—O2—C2—C1	17.4 (3)
Li1^i^—O1—C1—N1	104.1 (3)	C6—N2—C2—O2	4.6 (4)
Li1^ii^—O1—C1—N1	−121.1 (3)	C5—N2—C2—O2	177.4 (3)
Li1—O1—C1—C2	107.6 (4)	C6—N2—C2—C1	−173.7 (3)
Li1^i^—O1—C1—C2	−133.1 (2)	C5—N2—C2—C1	−0.9 (4)
Li1^ii^—O1—C1—C2	1.7 (3)	C6—N2—C2—Li1^ii^	−33.1 (8)
Li1—O1—C1—Li1^ii^	105.9 (4)	C5—N2—C2—Li1^ii^	139.7 (6)
Li1^i^—O1—C1—Li1^ii^	−134.8 (3)	O1—C1—C2—O2	−13.3 (4)
C3—N1—C1—O1	−71.2 (3)	N1—C1—C2—O2	114.8 (3)
C4—N1—C1—O1	53.4 (3)	Li1^ii^—C1—C2—O2	−12.1 (2)
C3—N1—C1—C2	164.3 (2)	O1—C1—C2—N2	165.0 (2)
C4—N1—C1—C2	−71.1 (3)	N1—C1—C2—N2	−66.9 (3)
C3—N1—C1—Li1^ii^	−119.0 (3)	Li1^ii^—C1—C2—N2	166.2 (3)
C4—N1—C1—Li1^ii^	5.6 (4)	O1—C1—C2—Li1^ii^	−1.2 (2)
Li1^ii^—O2—C2—N2	−160.9 (3)	N1—C1—C2—Li1^ii^	126.9 (2)

Symmetry codes: (i) *y*−1/3, −*x*+*y*+1/3, −*z*+1/3; (ii) *x*−*y*+2/3, *x*+1/3, −*z*+1/3; (iii) −*y*+1, *x*−*y*+1, *z*; (iv) −*x*+*y*, −*x*+1, *z*.

### Hexakis[µ3-1,2-bis(dimethylamino)-2-oxoethan-1-olato]hexalithium(I) (II) Hydrogen-bond geometry (Å, º)

**Table:**

*D*—H···*A*	*D*—H	H···*A*	*D*···*A*	*D*—H···*A*
C1—H1···O2^iv^	1.00	2.59	3.322 (4)	130
C5—H5*B*···N1	0.98	2.34	3.059 (4)	130

Symmetry code: (iv) −*x*+*y*, −*x*+1, *z*.
